# Hints of Biological Activity of Xerosydryle: Preliminary Evidence on the Early Stages of Seedling Development

**DOI:** 10.3390/ijms25168717

**Published:** 2024-08-09

**Authors:** Filippo Geuna, Andrea Pensotti, Raffaele Vecchione, Roberto Germano

**Affiliations:** 1 Department of Agricultural and Environmental Sciences—Production, Landscape, Agroenergy (DISAA), University of Milan, 20133 Milan, Italy; 2 Systems Biology Group Lab, Sapienza University of Rome, 00185 Rome, Italy; a.pensotti@unicampus.it; 3 Research Unit of Philosophy of Science and Human Development, Campus BioMedico University, 00128 Rome, Italy; 4 Center for Advanced Biomaterials for Health Care (CABHC), Istituto Italiano di Tecnologia, Largo Barsanti e Matteucci 53, 80125 Napoli, Italy; raffaele.vecchione@iit.it; 5 PROMETE S.r.l., CNR Spin off, Piazzale V. Tecchio, 45, 80125 Napoli, Italy; germano@promete.it

**Keywords:** xerosydryle, chlorophyll, transcriptome analysis, Nafion, exclusion zone (EZ), abiotic stress

## Abstract

Xerosydryle belongs to a new category of materials resulting from the interaction of water with various hydrophilic polymers. These materials can exhibit different properties depending on the kind of polymer-water interaction. Previous research confirmed the existence of a solid manifestation of water at room temperature. The thermal properties of dissolved xerosydryle in water are similar to those of biological macromolecules during denaturation but with greater stability. This study investigated the biological effect of xerosydryle on a living system for the first time, using a seed germination model. The interaction was evaluated using physiological assays such as chlorophyll shifts, potassium (re)uptake during the onset of germination and a transcriptome approach. Seeds were treated with samples of xerosydryle and distilled water. Transcriptome analysis of germinating seeds highlighted differences (up- and down-regulated genes) between seeds treated with xerosydryle and those treated with distilled water. Overall, the experiments performed indicate that xerosydryle, even at low concentrations, interferes with seedling growth in a manner similar to an osmotic modulator. This work paves the way for a more comprehensive exploration of the active biological role of xerosydryle and similar compounds on living matter and opens up speculation on the interactions at the boundaries between physics, chemistry, and biology.

## 1. Introduction

Iterative interactions between water and inert, hydrophilic polymers that cannot dissolve are common in various natural systems. Over the past decade, several studies have shown that large, supramolecular organized structures form upon such contact with water. A solid residue remains once the leftover water is subjected to freeze-drying after the removal of the polymer. The experimental results, published in a dozen international papers (e.g., [1,2,3]) indicate that the composition of this residue differs from the original material that came into contact with the water. Notably, the residue demonstrates exceptional stability against heat. Its thermal characteristics and infrared spectrum are influenced by the specific material that has been repeatedly in contact with the water. Depending on the type of material used, these residues can take the form of gels, powders, or even polymers. These residues represent a new category of materials that we label as “xerosydryle”. This word comes from the ancient Greek terms, “xeros” (dry), “hydro” (water), and “yle” (matter) [4].

Notably, Gerald H. Pollack and colleagues [5] advanced these findings when replicating our experiment. They showed that an exclusion zone (EZ) water resulted from contact with chemically different surfaces, such as Nafion and Whatman-5 filter paper. After the treatment, such water was analyzed using an ultraviolet-visible absorbance spectroscopy. Then, it was solidified via lyophilization or oven evaporation. The resulting solid structure displayed remarkable stability. Mass spectroscopy analysis revealed the absence of ionizable contaminants that can reproduce the characteristic “EZ signature” spectra observed in the three liquid preparations, as well as in the solids formed from dehydrated EZ water that had been reconstituted in deionized water. This collective evidence led to the independent conclusion, in alignment with the initial discoverers, that a solid manifestation of EZ water indeed exists at room temperature.

A previous work [6] offered a theoretical explanation of this phenomenon, where multiple indicators point towards its inherent quantum nature.

Of particular note is the observation that when xerosydryle is dissolved in water, it exhibits circular dichroism (CD). This property is linked to the interaction of polarized light with chiral molecules. Interestingly, our measurements indicate that the thermal characteristics of dissolved xerosydryle in water closely resemble those of biological macromolecules during processes akin to “denaturation”. However, these dissolved structures display a greater thermal resistance than biomolecules. Moreover, the chirality of xerosydryle remains unaffected even when sodium hydroxide (NaOH) or hydrogen chloride (HCl) are added in sufficient quantities to raise the pH to 13 or lower it to 3, respectively. This remarkable robustness and the observation that alterations in hydration can induce substantial changes in the DNA structure suggest potential connections to the DNA-repair mechanisms. The origin of biohomochirality—the prevalence of the same chirality in living organisms—remains a mystery. These findings propose that mirror-symmetry breaking within water might play a significant role in the emergence and prevalence of biohomochirality.

Additionally, these structures can revert to their previous physicochemical properties upon reintroduction into pure water, which is akin to the ability of certain simple living systems like bacteria or protists to enter a quiescent state when conditions become unfavorable. This process, known as encystment, allows them to resume their active growth when the environment becomes suitable (excystment).

The parallel between these structures and simple life forms is striking. Dissipative structures in pure water are aggregates of water molecules resulting from various types of perturbations, including low-energy ones. They can persist far from thermodynamic equilibrium for extended periods of several years or more. Once the bulk water is removed, much like simple lifeforms, they enter a “frozen” dormant state, halting energy dissipation until ample bulk water is newly available. Just as simple life forms transition from quiescence to activity, these structures have the capability to revert from a quiescent state to an active one, re-establishing themselves as dissipative structures that are far from equilibrium.

This information strongly implies the potential that these structures serve as the foundational framework for life itself. Hence, we decided to conduct the first ever study involving the biological activity of xerosydryle on a relatively simple experimental biological system. In this experimental work, we used the xerosydryle obtained by iterative contact with silk [4]. Further elaboration on this matter can be found in Appendix A.

Seed germination requirements for temperature and moisture have been well documented through thermal-time, hydro-time, and hydrothermal-time models [7,8,9,10,11]. Research on threshold values has been mostly conducted for agricultural and ecological purposes in different model species, as well as species of agronomic interest.

Several methods can be used to infer changes in plant seeds following exposure to chemical compounds, such as the visual inspection of germination [12,13], metabolite profiling [14,15], gene expression profiling [16], and Fourier transform infrared spectroscopy (FTIR) analysis [17].

In this work, we opted for a combination of physiological analysis and transcriptional profiling. As a source of experimental material, we chose fenugreek (*Trigonella foenum-graecum*) seeds since they prove to be a reliable species in investigating the physiology of germination and the interaction with experimental compounds [18,19,20,21]. Moreover, fenugreek seeds are small enough to provide a convenient size for management and sufficiently large to assure a self-sustained germination growth without the need for any external output over several days, given the peculiar testing condition.

## 2. Results

The aim of the present work was to verify whether xerosydryle produces any physiological effects in living systems. For this purpose, some physiological and molecular tests were devised by applying the substance in a water solution versus distilled water. This was the control. In particular, the tests involved the effect of the substance on the first stages of seed germination. Parameters such as chlorophyll profiles, weight development, and transcriptomic profiles were measured and evaluated.

### 2.1. Weight of Seedlings during Germination

**Figure 1:**
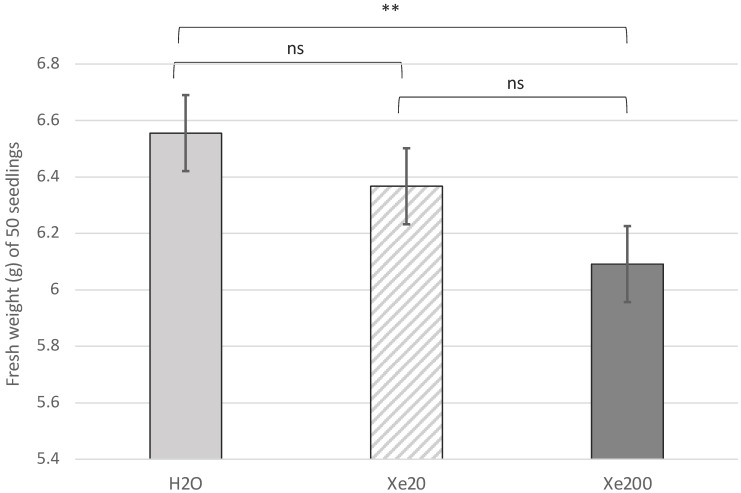
Analysis of variance (ANOVA) of seedlings grown in different conditions. Batches of 50 seedlings growing in distilled water (‘H_2_O’), xerosydryle 20 mg/L (‘Xe20’), and xerosydryle 200 mg/L (‘Xe200’) were weighed at 8 days after the onset of germination. Bars indicate the confidence intervals (CI 95%) as standard deviation. ns = not significant; ** = *p* < 0.01.

Weight data were recorded at eight days from the initiation of germination. Data were recorded from no less than three different experiments. In all cases tested, a similar trend was observed between the three conditions, where the distilled water condition showed the highest weight. Moreover, an ANOVA analysis followed by the least significant difference (LSD) *post-hoc* test revealed that the only significant difference was observed between distilled water (‘H_2_O’) and xerosydryle 200 mg/L (‘Xe200’) (Figure 1). Only one further experiment showed a deviation from this pattern with xerosydryle 20 mg/L featuring a slightly higher weight than distilled water alone.

### 2.2. Chlorophyll Content

**Figure 2:**
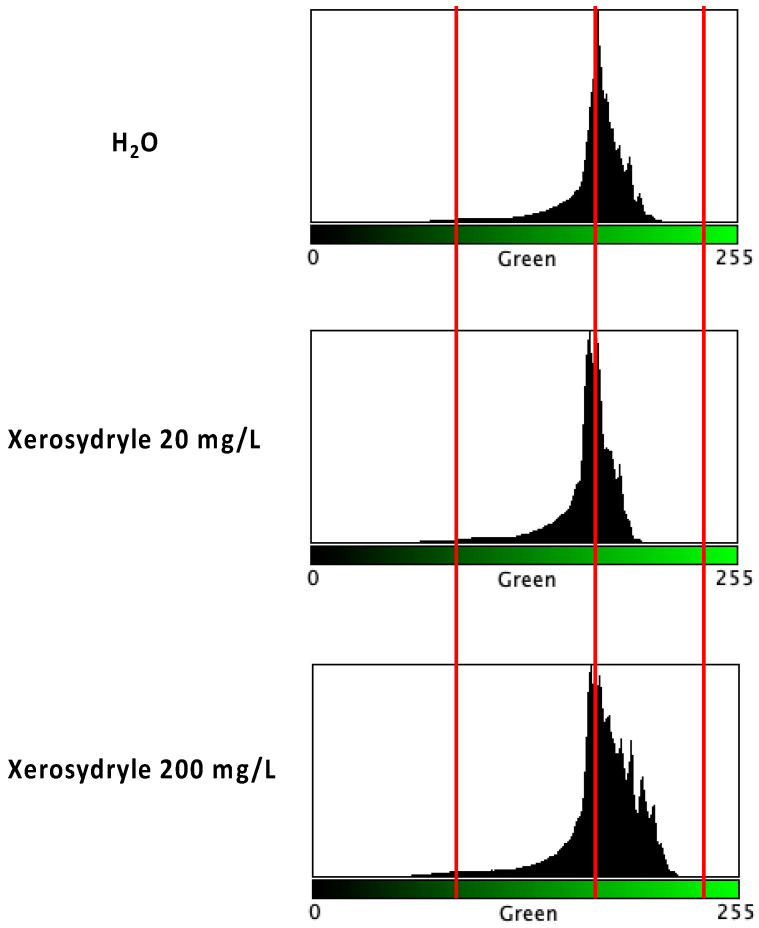
Image analysis of chlorophyll accumulation. After scanning 50 seedlings per treatment, an RGB color analysis was performed and the “green” channel was extracted for comparison. Vertical red bars highlight the profile differences between treatments and the shift in the histograms. The x-axis represents the values in the 8-bit scale of “green” levels. The y-axis represents the pixel count.

Measurements of chlorophyll accumulation were performed during at least three distinct experiments with seed germination over 8 days. Similar profiles were observed, showing a clear qualitative and quantitative shift in the green RGB channel histograms. A visual comparison of the resulting measurements is shown in Figure 2.

**Figure 3:**
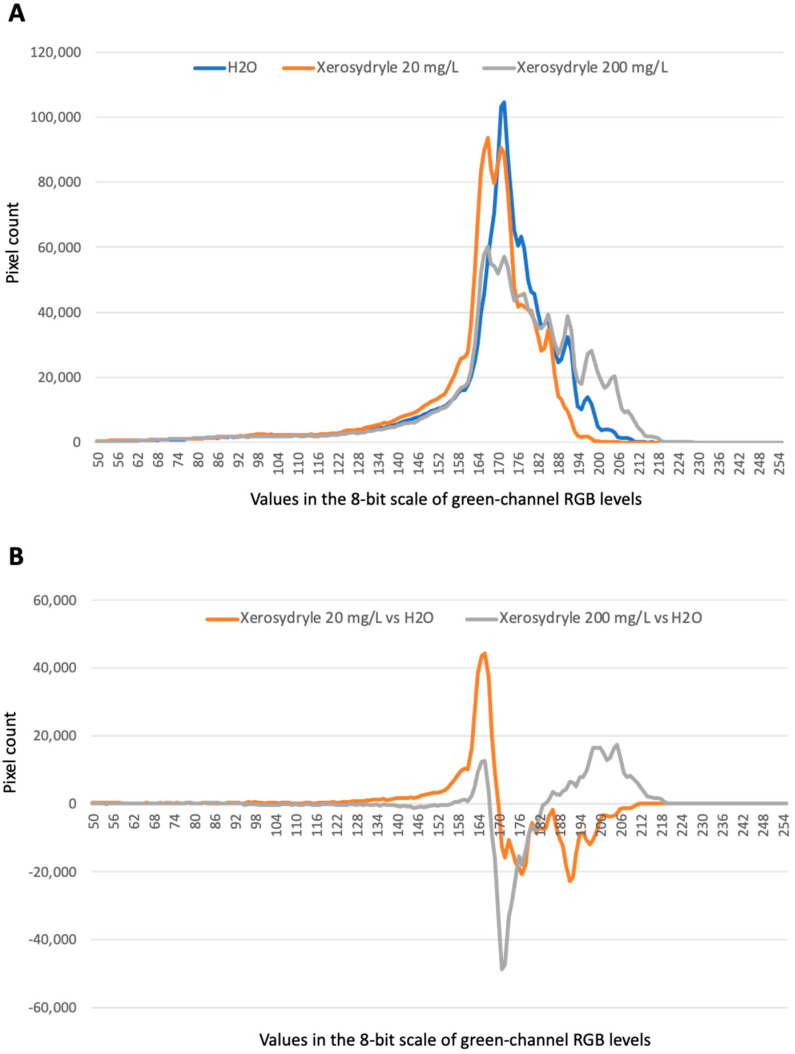
Comparative image analysis of chlorophyll accumulation. (**A**) The “green” channel of the RGB histogram for the three samples is plotted. (**B**) Point-to-point difference of the “green” channel values between xerosydryle 20 mg/L (grey line) and xerosydryle 200 mg/L (orange line) and water (baseline).

In all of the experiments, a shift towards brighter “green” tones was observed, especially at the 200 mg/L concentration of xerosydryle in comparison to water alone. A further comparison of the “green” RGB channel histograms under the three growth conditions is provided (Figure 3A). The superimposition of plots at a higher spatial resolution around the peaks clearly shows similar profiles. However, the xerosydryle 200 mg/L plot extends to brighter green tones with a concurrent reduction in the overall peak intensity. A comparison of the point-to-point differences between the two xerosydryle concentrations versus water is also provided with two major yet displaced peaks in the “green” RGB channel interval between 164 and 176 (Figure 3B).

The dynamic type warping (DTW) analysis produced a ‘normalized distance’ of 647.035 between xerosydryle 20 mg/L and water and a distance of 1177.265 between xerosydryle 200 mg/L and water. The graphical output of the analysis is also provided (Supplementary Figure S1).

### 2.3. Effect of Xerosydryle on Potassium Release during Seed Germination

**Figure 4:**
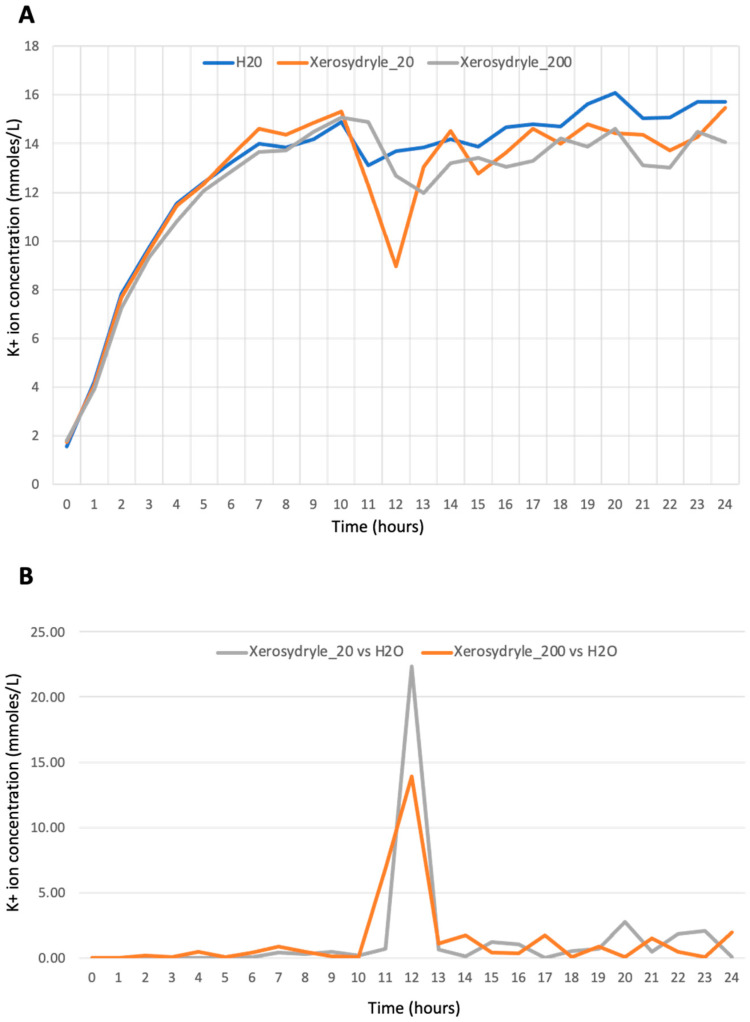
K measurement at the early stage of germination. (**A**) Profile of K release and reuptake during the first 24 h of germination. Blue line: H_2_O. Orange line: xerosydryle 20 mg/L. Grey line: xerosydryle 200 mg/L. (**B**) Data normalization of K^+^ concentration of xerosydryle 20 mg/L vs. H_2_O (grey line) and xerosydryle 200 mg/L vs. H_2_O (orange line) (see Section 4).

A set of experiments on potassium (re)uptake was performed by comparing the two concentrations of xerosydryle with water (Figure 4A). The scope of this experiment was to assess whether xerosydryle can affect the early physiological stages of seed germination.

While the plots look similar in the first 10 h after the onset of germination, there is a clear difference in the interval between 11 and 12 h. This is when the xerosydryle 20 mg/L samples show a faster reduction compared to both water alone and xerosydryle 200 mg/L. The plots obtained were compared using the metric described (Section 4), which produces total relative distances between xerosydryle 20 mg/L and 200 mg/L concentrations versus water of 35.94 and 33.76, respectively (Figure 4B). In particular, it can be noted that the maximum difference between both xerosydryle concentrations and water occurs around 12 h after the beginning of the experiment, when K^+^ concentrations start to lower following the rearrangement of seed cell membranes. At this point, which is diagnostic of possible physiological effects of the compound, xerosydryle 20 mg/L and 200 mg/L differ from the water of 22.36 and 13.91, respectively. The water of 22.36 exhibits a significant difference, as determined by a *t*-test.

### 2.4. Transcriptome Analysis

**Figure 5:**
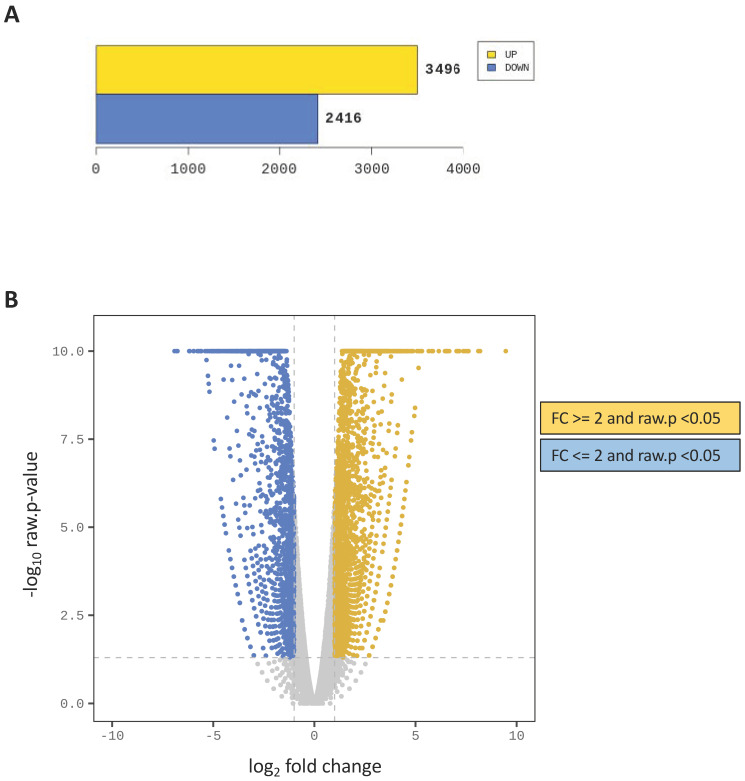
Fold change analysis of transcriptome data. (**A**) Up- and down-regulated transcripts between xerosydryle 200 mg/L and H_2_O with absolute values of fold change (FC) ≥ 2 and *p* < 0.05 are shown in yellow and blue, respectively. (**B**) Volcano plot representation of the fold change data.

A comparative transcriptional (RNA-seq) analysis between seedlings grown in distilled water and xerosydryle 200 mg/L, respectively, was performed. For the seeds grown in distilled water, 21,456,158 reads were generated corresponding to 2,167,071,958 total base sequences with a Q30% of 93.82. For seeds grown in xerosydryle, 200 mg/L 23,587,524 reads were generated corresponding to 2,382,339,924 bases with Q30% of 94.16. First, reads from the two samples were aligned to gene databases to reconstruct a de novo fenugreek transcriptome of 65,144 contigs which, after filtering and TMM normalization, yielded 41,232 contigs for the two samples. These data were then used to perform a differential gene expression (DEG) analysis that showed a total of 3496 up-regulated and 2416 down-regulated genes (DEGs) between xerosydryle and distilled water (Figure 5).

The complete list of unigene data and the corresponding gene ontology (GO) information is available in Supplementary File S1 and Supplementary Figure S2. Ordering the list of differentially expressed genes according to the fold change value (positive to negative) allows for retrieving the genes that show the highest fold changes in both up and down-regulation. Contigs with negative fold change values in the comparison of xerosydryle versus distilled water correspond to genes that are over-expressed in distilled water. Conversely, contigs showing positive fold change values represent genes that feature a higher expression in xerosydryle than in water.

## 3. Discussion

Different types of evidence, physiological and transcriptional, appear to confirm a biological effect of xerosydryle by its effect on seed germination and the early stages of seedling growth. Curiously, some of the various tests performed in the present research, namely the weight of seedlings during growth and the K+ reuptake, seem to prove that a lower concentration (20 mg/L) of this compound has a greater effect than a higher concentration (200 mg/L).

The data presented here, which are far from being exhaustive, suggest further investigation. In particular, this involves assessing the effect of a broader range of xerosydryle concentrations and the possible role of other factors like the dependence on temperature, radiation, and possible physicochemical changes that occur at the interface with the biological system. In fact, in this work, we decided to compare only the higher xerosydryle concentration (200 mg/L) against water at the transcriptome level. A further investigation on the 20 mg/L and possibly other concentrations should be undertaken in the future. Moreover, distinct classes of xerosydryle-like compounds were obtained. These were characterized by a range of different physicochemical properties. It would therefore be interesting to test their respective effects at the biological level.

The reuptake of K is a physiological consequence of the rearrangements of cell plasma membranes that undergo restructuring in the first phases of seed germination [22,23]. The differences are shown by comparing the two concentrations of xerosydryle and distilled water parallel those observed in the other physiological tests. Once again, it demonstrates that the lower xerosydryle concentration (20 mg/L) affects seed germination (through K+ uptake) in a slightly more marked yet statistically significant way than the higher concentration (200 mg/L).

Strong evidence of the interaction of xerosydryle and living matter is provided by transcriptome data that show a consistent modulatory effect on multiple genes and metabolic processes. The structure of Gene Ontology (GO) terms is such that multiple descriptors are usually assigned to each gene in a transcriptome study. The enrichment of a particular group of terms is thus diagnostic of specific metabolic changes occurring in the cells and tissues under examination. In particular, the GO analysis revealed a set of prevailing terms under the ‘Biological process’ (BP), ‘Cellular component’ (CC), and ‘Molecular function’ (MF) subgroups. For the CC subgroup, the first three terms in decreasing order of occurrence are ‘GO:0044464 cell part’ (3001 counts), ‘GO:0043226 organelle’ (1908 counts), and ‘GO:0016020 membrane’ (955 counts). Interestingly, they point to the involvement of transcriptional changes that affect the cell compartmentation network of organelles and membranes, with organelles being central in energy metabolism through the production of photosynthetic pigments and the activity of photosystems.

The contig with the highest negative fold change (xerosydryle treatment versus water) is type I inositol 1,4,5-trisphosphate 5-phosphatase 2-like (IP5PII), a gene involved in inositol signaling metabolism. The complex interplay between different actors in the inositol signaling mechanism has been mainly studied in *Arabidopsis thaliana* and some other model species. Interestingly, under abiotic stress, there are several known cross-talks between the inositol signaling pathway and phytohormones, notably abscisic acid (ABA). Moreover, the mechanisms that underlie several physiological responses to stress stimuli, including salt and osmotic stress, are governed by the modulation of the inositol metabolism [24].

The second contig with the highest negative fold change appears to be a plant cationic peroxidase 1. Peroxidases are known to play a role in several metabolisms that range from oxidation of toxic reductants, the removal of H_2_O_2_, biosynthesis and degradation of lignin, suberization, auxin catabolism, response to environmental stresses such as wounding, pathogen attack, and, notably, oxidative stress [25].

Also, the gene coding for a leucine-rich repeat (LRR) extensin-like protein 6 is highly down-regulated. Plant cells require a unique set of regulatory mechanisms in order to modify the extracellular matrix. The cell wall protects against biotic and abiotic stresses and, importantly, determines the shape of each cell. Leucine-rich repeat (LRR) extensins (LRXs) are cell wall-localized chimeric extensin proteins and *LRX6* is expressed during lateral root formation [26,27]. Several sorts of interactions form the base of the construction of a cohesive structure made of wall polysaccharides, including noncovalent binding interactions, covalent bonding, and physical entanglements. Noncovalent interactions between wall polymers are so abundant that the growing wall may be considered a structure based largely on supramolecular chemistry [28].

The fourth contig for fold change corresponds to a glutathione S-transferase (GST), one of nature’s most versatile enzymes. It catalyzes a wide range of reactions that involve the conjugation of glutathione (GSH) to electrophilic compounds so that more soluble peptide derivatives can be formed. The GSTs are involved in response to oxidative stress, including drought, salt, and heavy metals [29].

The fifth contig codes for Responsive to Desiccation protein 22 (RD22), a typical ABA-responsive gene, is known for the role of abscisic acid (ABA) in drought and salinity stress signaling [30].

Among the contigs showing positive fold change values, the highest ranking one (fc = 705,71) codes for an elongation factor 1 A (EF1A) protein. The EF1A, an essential regulator for protein synthesis, has been reported to participate in abiotic stress responses and environmental adaptation in plants. A total of 34 EF genes were identified in the *Medicago truncatula* genome, a model species similar to fenugreek. In particular, an expression analysis result in response to salt treatment showed that MtEF1A1 was induced by salt stress in multiple tissues and that the expression level increased significantly after 8 h. A model was proposed for the regulatory mechanism of MtEF1A1 in response to salt stress. Salt stress induces the expression of MtEF1A1 through Ca^2+^ signaling. The MtEF1A1 promotes the expression of calmodulin (CaM), which in turn binds Ca^2+^ to trigger the expression of downstream genes [31] and references therein).

The second most expressed unigene codes for a protein related to the ribosome structure and functions. Ribosomal S3 family protein is a structural constituent of ribosome, which is recognized as being involved in response to salt stress, translation, and response to abiotic stimuli in general [32].

The experimental data show how the effects produced by xerosydryle on seed germination and the first stages of seedling development are comparable with those of other chemical compounds that modulate germination like, for example, salinity stress produced by NaCl [33,34]. For potent germination modulators with hormone-like effect, the range of tested concentrations can be lower as, for example, in the case of the inhibitory effect of abscisic acid (ABA) on *Arabidopsis* germination [35]. The transcriptome analysis in this work highlighted many genes involved in the salinity stress response. The contig showing the highest fold change in xerosydryle 200 mg/L-grown seedlings resulted in an elongation factor 1 A (EF1A) protein, which is known for its role in the physiological response to salt stress. The tremendous activation that it shows in the presence of xerosydryle clearly correlates the latter with salt stress and poses the basis for a wider set of investigations using this gene as a marker of response to xerosydryle application on plants.

Several abiotic and biotic factors are known to have a marked impact on seed germination [36]. They can delay, reduce, and also prevent germination. For instance, salinity is a factor that limits not only plant growth but also seed germination [17]. It reduces the plant’s capacity for water absorption, which triggers several physiological and metabolic processes that cause the prolongation of seed germination time. This mainly happens by increasing the osmotic pressure. Furthermore, seed germination periodicity and timing are mostly affected by temperature [8]. The germination rate increases with temperature proportionally up to its optimum, then decreases sharply. Likewise, soil pH is also reported as one of the factors that significantly influence seed germination [37].

It is known that leaf chlorophyll content is a key indicator of the physiological condition of a plant. The excess amount of reactive oxygen species (ROS) is scavenged by the enzymatic and nonenzymatic components of the plant cell’s antioxidant defense. Nevertheless, when the rate of ROS generation overwhelms the detoxifying potential of the plant, it leads to oxidative stress. Ultimately, this inflicts damage on several molecular species, including proteins, DNA, lipids, and the photosynthetic apparatus. The effect of salinity stress on chlorophyll content is well-known in many plant species [38,39]. Color data obtained from digital imaging can, in fact, be used for the rapid, non-destructive, and accurate estimation of chlorophyll content and as a measure of plant health [40,41,42]. In the present work, several genes featuring a differential expression at the transcriptome analysis were involved in the cascade of ROS-mediated events. This can explain the photosynthetic pigment accumulation observed as lighter tones in the green channel of the RGB spectrum [43].

The role of water in biological systems, still far from being fully understood, deserves special attention and reconsideration. In fact, the subject is undergoing a paradigm shift at the intersection of biology, chemistry, and physics. Too many phenomena associated with the physicochemical properties of water are largely unknown or still poorly addressed. For example, the understanding of redox and photochemical reactions in aqueous environments requires a precise knowledge of the ionization potential and electron affinity of liquid water. While the former has been measured, the latter has been addressed rather recently. Knowledge of the water electron affinity is key in understanding mechanisms of redox reactions in aqueous systems that involve either molecular species or solid surfaces [44]. Current cell biology considers water as a mere background carrier of the more important molecules of life. Nevertheless, water may be considered a central player in life processes. Water is known for having three phases (gas, liquid, and solid). However, over recent years, the presence of a surprisingly extensive “fourth phase” that occurs at interfaces has been demonstrated. The formal name for this fourth phase is exclusion zone (EZ) water [45,46].

From the plant biology perspective, the fact that xerosydryle is generated by the iterative exposition of pure water to polymers like cellulose is particularly intriguing. This is because the plant cell wall itself is built on polysaccharides, and the intricate interplay between synthesis and degradation occurs at the interface of water and polymers [28].

The body of experimental evidence produced in this work clearly testifies for the first time that xerosydryle has a biological effect. This allows for speculations on the biological role of, for example, iteratively filtered water—something extensively and spontaneously happening in nature—and its coherent deformed states [1,46]. Further studies are needed to determine at the molecular and biophysical levels how xerosydryle and similar substances may influence and possibly explain the mechanisms that underlie the physics of living systems.

We are aware that data from [47] has led the authors to hypothesize a certain amount of contamination in the iteratively perturbed water samples derived from Nafion sheets, compatible with a possible microbiological origin. Consequently, they elaborate on the nature of iteratively perturbed water (xeroydryle) as the result of an alleged measurement artifact. However, the mass spectroscopic analysis performed by [48] on the solid residue (see Figure 2 therein) clearly indicates the water signature. On the other hand, these last EDX-based analytic results on the solid material (xerosydryle) counter the speculation on the “contamination” hypothesis” and confirm the results of [48].

We are confident that our measurements are correct, although, considering the caution of the academic editors of this article, further research from independent laboratories would further rule out any doubt on the nature of this class of compounds, while promoting a constructive scientific debate.

## 4. Materials and Methods

### 4.1. Plant Material

Seeds of fenugreek (*Trigonella foenum-graecum* L. family Fabaceae, http://www.theplantlist.org/tpl1.1/record/ild-8021; accessed on 20 February 2023), obtained from Fresh Tropical S.r.l. (Paderno Dugnano, Italy) were soaked in distilled water for 24 h before being transferred into 9 cm-diameter round glass vials covered by a Petri dish’s glass cover. 50 seeds were used per treatment. As a comparison, 50 seeds were also placed individually in 50-well polypropylene vials filled with growth liquids.

### 4.2. Chemicals

The water used for the experiments was of pharmaceutical-grade purity according to ISO 9001 2015 (Idrochimica, Pieve Emanuele, MI, Italy, lot n. 131021).

Promete S.r.l. (Naples, Italy) produced and supplied xerosydryle in dried form according to the protocols in [2,3,4]. We let Milli-Q^®^ water repeatedly touch raw silk (kindly provided by Antico Opificio Serico, Caserta, Italy). In other terms, we washed raw silk in Milli-Q^®^ water and dried it in the air. After that, we repeated the following procedure 10 to 50 times: we submerged raw dried silk in Milli-Q^®^ water, which was contained in an open polystyrene Petri dish, gently stirring the liquid, measuring its electric conductivity, and reshuffling the silk. We then removed the raw silk and dried it in the air. The variation depends on not only the number of iterations but also the volume of the sample and the time of hydration. For this reason, we speak of a range (10–50 times) and not an exact number. After the silk was taken out, we labeled it “iteratively perturbed water (IPW)”. The IPW samples were typified by their electric conductivity. In fact, measuring this value was simple and did not destroy or pollute the specimen. Each reshuffle of the polymer enhanced conductivity. The enhancements surpassed the experimental error after several shuffles. Finally, lyophilization of the modified water led to solid xerosydryle. Xerosydryle was dissolved in distilled water at the concentrations required for treatments as needed. Solutions were stored at room temperature in the dark until use.

### 4.3. Germination Treatments

Round glass vials (diameter: 10 cm; height: 4 cm) containing 50 seeds added with 5 mL of water or xerosydryle dissolved in water at the final concentration of 20 mg/L (0.5 mg/25 mL) or 200 mg/L (5 mg/25 mL), respectively, were placed under a light-emitting diode (LED) (200 μmol m^−2^ s^−1^) for 14 h/day at 22 °C. Evaporation and microbial contamination were counteracted by adding water every two days and keeping the seeds covered. Waterlogging was also prevented by keeping the liquid coverage at a minimum.

### 4.4. Potassium Reuptake Measurement at Germination

Fifty seeds were placed into 50 mL Erlenmeyer flasks and covered with 5 mL of test solutions (double-distilled water or xerosydryle dissolved at the two different concentrations described above). Flasks were kept in agitation at 90 rpm on a shaker at 22 °C and constant illumination during the entire experiment, as previously described. One hundred microliter samples were collected every hour and placed into polypropylene microtubes. These were diluted in 5 mL of 2% nitric acid prior to reading. This was performed with a model 7850 ICP-MS mass spectrometer equipped with a model SPS 4 Autosampler (Agilent Technologies, Santa Clara, CA, USA). Readings are expressed as millimoles/L.

The raw readings from the three samples were imported into a Microsoft Excel spreadsheet and used to generate plots. Readings were further compared in a pairwise mode following the metric: sum([K^+^]_xerosydryle XX mg/L_ − [K^+^]_H2O_)^2^), which sums up, point by point, the difference of concentration of K^+^ released in the xerosydryle-treated sample (20 or 200 mg/L, respectively) against the water control. The distances are expressed as unitless values [49].

### 4.5. Measuring Seedlings Weight

Weight data were recorded from lots of 50 seeds in distilled water, xerosydryle 20 mg/L, and xerosydryle 200 mg/L, respectively. Temperature was kept constant at 22 °C, and a 12-h photoperiod was maintained during the entire experiment. When necessary, the liquids were completely aspirated from growth vessels, and the seedlings were carefully moved to blotting paper and briefly dried before being weighed with a balance to three decimal places. If requested, seedlings were returned to their vessels and fresh growth liquids were restored to their original conditions to continue growth.

Statistical differences were estimated by the one-way ANOVA test (Excel v. 16.66.1 for Mac) using a *p*-value threshold of 0.05 for at least three repetitions [50]. This was followed by the least significant difference (LSD) post-hoc test.

### 4.6. Chlorophyll Content Determination through Image Analysis of Germinating Seedlings

Chlorophyll content was measured in a non-destructive way following a modification of the method described by [40]. Samples of 50 seeds germinated under the conditions described above for a period of 6 to 8 days in the presence of water alone or xerosydryle at different concentrations. At the time of measurement, seedlings were collected from the vials, blotted on dry paper to ensure the removal of any residual liquid, and placed, well separated, on a Stylus SX125 (Epson) scanner glass plate for image acquisition. Alternatively, images were taken using a color digital camera.

Images were acquired through the scanner via Image Acquisition v. 8.0 software running on a MacBook Pro (Apple). They were obtained at a resolution of 150 dpi in RGB mode and its built-in internal white cover or with a red paper sheet to enhance the contrast of plant material. The NIH ImageJ2 (“Fiji”) v. 2.9.0/1.53t software (https://imagej.net; accessed on 10 March 2023 [51]) running on the same computer was used to analyze images.

Histograms of pixels were obtained through the ‘Analyze→Histogram’ command of the Fiji software. The “Green” channel was selected by toggling the “RGB” button in the corresponding window and was chosen to represent the accumulation of photosynthetic pigments (mostly chlorophylls). The resulting data were copied from NIH ImageJ2 software and pasted into Microsoft Excel for further elaboration. At this stage, graphs of RGB pixel histograms were plotted by combining the values of the different treatments.

Chlorophyll accumulation in control seeds germinated at different xerosydryle concentrations and control water was assessed by comparison of histograms of the “green” channel readouts. The point-to-point difference in the “green” channel values between xerosydryle 20 mg/L (gray line) and xerosydryle 200 mg/L (orange line) and water was calculated.

The resulting plots were compared following the Dynamic Time Warping (DTW) statistical analysis method ([52]; https://dtw.r-forge.r-project.org/). The DTW is an algorithm used to measure the similarity between two sequences. First, a matrix of the Euclidean distances between each pair of points from the two sequences is calculated. Among these distances, different warping paths can be found, meaning the possible stretching of different sequences to become as similar as possible. The method quantifies the similarity between sequences by finding the best warping path, which corresponds to the one with the smallest accumulated distance. The similarity between two or more sequences can be quantified with the resulting distance, that is, the accumulated Euclidean distances of the path.

### 4.7. Transcriptome and Differential Gene Expression Analysis

A whole-transcriptome analysis was performed between seedlings grown in distilled water and xerosydryle 200 mg/L in the above-mentioned conditions. The solutions were replaced every two days for a total period of 8 days. The plant material, resulting from bulks of 50 seedlings at 6 days of development in the presence of distilled water or water and xerosydryle, was collected in 50 mL plastic centrifuge tubes, snap frozen in liquid nitrogen, and immediately stored at −80 °C until shipment in dry ice to the laboratory (Macrogen, Seoul, Republic of Korea) for RNA extraction, library preparation, and next-generation sequencing.

The total RNA was extracted using a Trizol [53] protocol. A quality assessment was performed using Qubit 2.0 and quantified with Bioanalyzer 2100 prior to library construction. Libraries were generated using the TruSeq Stranded mRNA Library Prep Kit (Illumina, San Diego, CA, USA). These were sequenced, and a de novo was assembled in two independent runs. In both runs, a multiplex approach was used to load the corresponding samples in the flow cells. A total of 1,165,201,460 paired-end 100 bp reads were obtained from both assemblies. Illumina adaptors were trimmed using Trimmomatic, and a quality assessment was performed using FASTQC. Reads were also trimmed to 75 bp to ensure a minimum quality score of Q20 in all bases, and low-quality reads were removed.

A de novo transcriptome assembly was performed in order to reconstruct the transcript sequences without a reference genome sequence. Assembled contigs are represented as the expressed transcripts for the species. Contigs were merged into non-redundant, unique transcripts that were as long as possible and then clustered into unigenes (unique genes) that had a minimum length of 200 bp.

To annotate the clustered unigenes, they were aligned against public databases with BLASTN and BLASTX, the Kyoto Encyclopedia of Genes and Genomes (KEGG), NCBI Nucleotide (NT), Pfam, Gene ontology (GO), NCBI non-redundant Protein (NR), UniProt, and EggNOG [54]. The prediction of the open reading frames (ORFs) was also performed to identify protein-coding regions within the unigenes. The unigenes were processed for read alignment, and their abundance was extracted as read count from the alignment. For each unigene, statistical analysis was performed to identify differentially expressed unigenes.

A bioinformatic analysis to compare differentially expressed genes (DEGs) was performed at Macrogen (Seoul, Republic of Korea). Raw data were deposited at the Sequence Read Archive (SRA) at the NCBI under the BioProject ID: PRJNA1059235.

The goal of pre-processing is to reduce systematic bias to avoid statistically erroneous conclusions by filtering, transforming, and normalizing data.

In order to reduce systematic bias, size factors were estimated from the read count data (calcNormFactors method). Using them, the read count data was normalized with the “Trimmed mean of M-values (TMM)” method in the edgeR package [55]. Then, a statistical test was performed with the normalized data.

The ‘log2(Counts per Million reads (CPM) + 1)’ and ‘log2(TMM normalized count + 1)’ values were used for data visualization. To prevent a negative value resulting from logarithm transformation, 1 was added to CPM (Counts per Million reads) or a normalized read count. These logarithm figures were used only for visualization. To proceed with a statistical test, the TMM normalized count was adopted for an exact test (‘exactTest’) in edgeR.

Significant contigs that satisfy the fold change and *p*-value (‘|fc| ≥ 2 & raw. *p* < 0.05’) conditions are provided (Supplementary File S1). The Gene Ontology (GO) descriptors associated with the contigs are also reported.

## Figures and Tables

**Figure A1:**
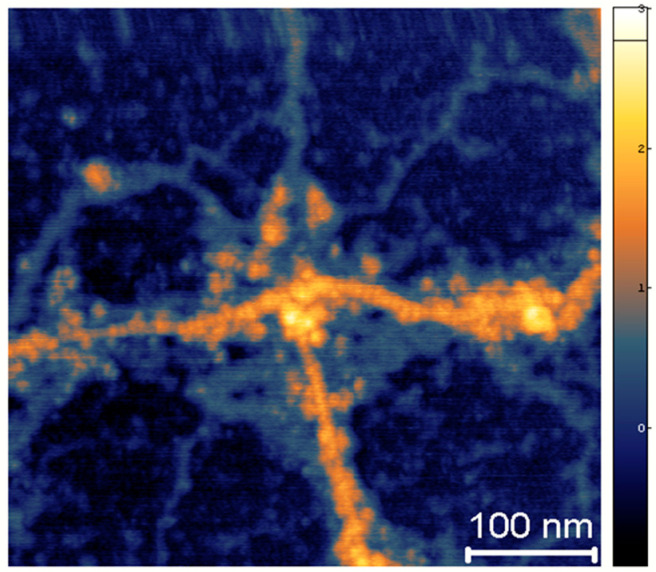
Topographic Atomic Force Microscopy (of perturbed water at 100 nm scale. The false color bar expresses the height in nanometers [60].

**Figure A2:**
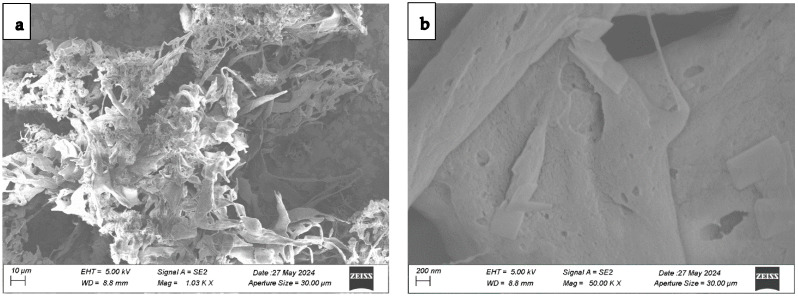
SEM images at two different magnification values of a lyophilized sample were obtained by perturbing 250 mL of Milli-Q^®^ water, repeatedly exposed to raw silk. Bars correspond to (**a**) 10 μm and (**b**) 200 nanometers.

**Figure A3:**
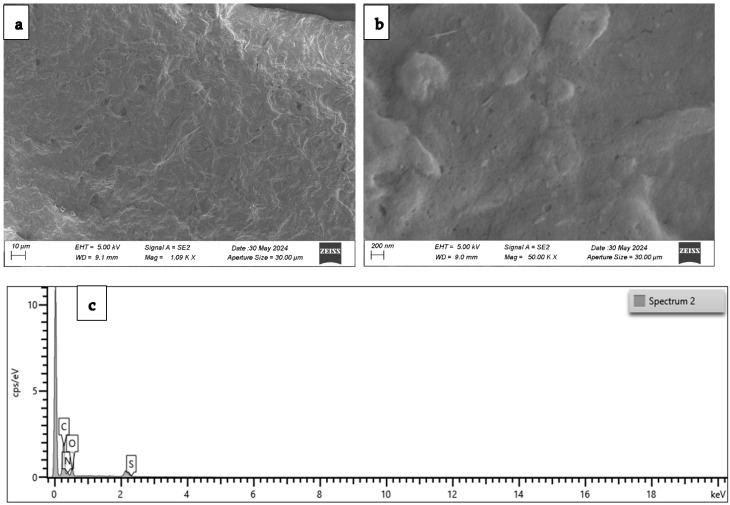
SEM images at two different magnification values of a sample obtained by redispersion in Milli-Q^®^ water, deposited on an aluminum stub, and sputter coated with gold. (**c**) EDX pattern of the same image displaying all the peaks including impurities and metals deriving from stub and metal coating. Bars correspond to (**a**) 10 μm and (**b**) 200 nanometers. The X-axis represents the energy while the Y-axis corresponds to counts per second per electron-Volt.

## Data Availability

The next-generation sequencing (NGS) reads for the transcriptome analysis were deposited in the Short Read Archive (SRA) database of the NCBI under BioProject ID: PRJNA1059235.

## Supplementary Materials

The following supporting information can be downloaded at https://www.mdpi.com/article/10.3390/ijms25168717/s1.

## Appendix A. The Chemical Nature of Xerosydryle

### Appendix A.1. Production Techniques of Xerosydryle

On page eleven of our paper, we dedicate sixteen rows to discussing how we produced this specific material in dried form, according to the protocol of the cited papers [1,2,3]. The material was obtained by letting Milli-Q^®^ water repeatedly touch raw silk a few dozen times. Moreover, in the following paper, cited in the manuscript: [4], we discussed the production of xerosydryle in general, and in particular its production from silk. In detail, under paragraph 4.2 that we report integrally here, xerosydryle obtained by silk (‘S’), which falls in the category of the fiber polymers, is highlighted in bold:

“We rinsed a polymer five times in 20 mL Milli-Q^®^ water. We dried the polymer in the air. We submerged the polymer in 10–50 mL Milli-Q^®^ water. The water was contained in a bowl that was not made of a hydrophilic material, e.g., a Petri dish, polystyrene, or plastic bowl. In the case of a sheet-shaped polymer (CE, PF, N), we mechanically, manually, or with a magnet, stirred the fluid. We only lightly stirred the fluid so that 2–3 mm of it rippled against the sheet. In the case of a fiber polymer (CA, S, SW, CR, HC), after ~15–30 min we mildly squeezed it with our polyethylene gloved hand. We took 1 mL of the fluid, measured its χ, and put the fluid back in the bowl. We gently moved the polymer. We repeated this sequence of stirring 20–50 times (or after each ~15–30 min squeezing), measuring chemical conductivity (χ), and moving the polymer. We took out the polymer and dried it in the air. After ~12 h, we put the polymer back in the bowl. We repeated tens of times this series of polymer submergence, stirring (squeezing), moving the polymer, drying the polymer, and χ measurement of the fluid. χ increases after each series. The recurrent contacts required for obtaining statistically significant variations in χ, i.e., variations beyond the experimental error, depend on the amount of the fluid and the amount of polymer employed. For example, for a water/SW (in grams) ratio of ~41: after the first recurrent contact series, χ = 22 μS cm^−1^; after the day 2 series, χ = 46 μS cm^−1^; after the day 3 series, χ = 76 μS cm^−1^; after the day 4 series, χ = 116 μS cm^−1^; after the day 5 series, χ = 131 μS cm^−1^. The fluid left in the bowl after the last polymer removal step was labeled IPW”.

We need to remind that dozens of international papers on the topic of xerosydryle have been published and iteratively perturbed water, and in each of them the preparation was explained, along with all the analyses showing that it cannot be confused with contaminants topic [2,3,6,48,56,57,58,59,60,61,62,63]. Moreover, it is important to mention that our recent research is the natural experimental progress of the work described in about 50 previously internationally published papers by Vittorio Elia’s research group and their collaborators. These papers provide evidence of the roots of this physicochemical phenomenon, which is observed by perturbing pure liquid water and is demonstrated by the generation of supramolecular H_2_O structures in water [64,65,66,67,68,69,70,71,72,73,74,75,76,77,78,79,80,81,82,83,84,85,86,87,88,89,90,91,92,93,94,95].

Finally, we would like to point out that our general experimental findings were independently replicated by Gerald. H. Pollack et al. [5]. In this study, the exclusion zone of water (EZ water) formed against three chemically distinct surfaces, and Nafion, ghee, and Whatman-5 filter paper were extracted. This extraction was performed by UV–visible absorbance spectroscopy and solidified either by lyophilizing or evaporation in an oven. The resulting, highly stable solid was analyzed by mass spectroscopy, which verified the absence of any ionizable contaminants that could reproduce the characteristic “signature EZ” spectra in the three liquid preparations, or in the solids formed from desiccated EZ water that had been reconstituted in deionized water. Therefore, based on this research, we concur that a solid form of EZ water indeed exists at room temperature.

### Appendix A.2. Chemical Analysis of the Solid Sample, Which Should Make It Possible to Derive a Chemical Formula for This Solid-State Water Polymer

As can be deduced from the Atomic Force Microscopy (AFM) topography described by [60], the basic component of the xerosydryle is nanometric (Figure A1). Nevertheless, it is very difficult to determine the chemical nature of this class of materials. As of this date, we cannot make a conclusive hypothesis on the chemical bonds.

Moreover, as explicitly requested by one of the reviewers of this paper, we performed a scanning electron microscope (SEM) investigation of a sample obtained by perturbing 250 mL of Milli-Q^®^ water via repeated contact with raw silk (the same sample used in the present work).

In the case of direct SEM analysis of the lyophilized sample, what appears is an intricate filamentous structure, possibly resembling a polymer. Below, an example of SEM on a lyophilized sample fixed on the stub by a carbon adhesive layer is reported (Figure A2a,b).

Subsequently, an energy-dispersive X-ray (EDX) analysis was performed. In order to avoid the introduction of other sources of carbon, the sample was first re-dispersed in Milli-Q^®^ water, then spotted and dehydrated directly on an aluminum stub and coated with sputtered gold.

The SEM images at two magnification values using a FESEM Ultra-plus (Zeiss) scanning microscope at 10 kV accelerating voltage are reported (Figure A3a,b).

In the same Figure A3c, it is also reported the energy dispersive X-ray (EDX) pattern displays several peaks. After excluding the aluminum peak of the stub, the gold peak of the coating, and some impurities, we focused on carbon, nitrogen, oxygen, and sulfur. In the following table, the list of their percentage weights are reported.

**Table A1:** Elemental analysis through energy dispersive X-ray (EDX) technique.

Element	Weight (%)
C	61.69
N	17.6
O	18.47
S	2.24

There is a consistent amount of carbon, and probably a large part of it comes from the atmosphere (“Process and apparatus for the capture and storage of the carbon of CO_2_ in the structure of the Xerosydryle”, Italian Patent pending: 102022000020472).

For the first time in the class of xerosydryle materials, we also found a large percentage of nitrogen, plus some sulfur. This interesting and specific result deserves further investigation because of its implications for understanding the mechanisms of organic molecule formation, thanks to the self-organizing action of water in its form of xerosydryle.

Further experiments are underway.

### Appendix A.3. Why the First Quantization (Hydrogen Bonding-Addressed by Reviewer #1) Is Inadequate to Justify Its Existence and Why the Second Quantization Is More Suitable

To explain this, we can refer to the following papers [6,96]. The emergence of a condensed, liquid phase at room temperature is a result of boson condensation, as described in the context of spontaneous symmetry breaking, so for a more accurate and authentic description of water, it is a transition from a semi-classical Quantum Mechanical perspective in the first quantization to a Quantum Field Theory perspective within the framework of second quantization.

The so-called “hydrogen bond” is a consequence of these dynamics, not the cause.

The emerging picture suggests that above a certain density threshold and below a critical temperature, the lowest energy state of a group of molecules interacting with their radiative electromagnetic field (EMF) is no longer a configuration where the oscillations (phases) of the molecules are uncorrelated and the EMF is negligible. Instead, it becomes a configuration where all molecules within a specific spatial region—called a coherence domain (CD)—have their phases synchronized, in tune with a non-vanishing EMF field trapped within the CD. The wavelength of this EMF determines the size of the CD. The CD acts as a cavity for the EMF field because the dynamics impart an imaginary mass to the photon, as described by the well-established Anderson–Higgs–Kibble mechanism. Within a CD, all molecules exhibit a larger volume than in their ground state because they oscillate in unison between their individual ground state and an excited state. This electrodynamic attraction is counteracted by thermal collisions, which disrupt the synchronization of the molecules. Thus, at a non-zero temperature T, similar to Landau’s model of liquid helium, each liquid becomes a two-phase system: a fraction Fc(T) of the constituent particles behaves coherently, while a fraction Fnc(T) = 1 − Fc(T) forms a dense gas trapped among the CDs.

These factors appear to be central mechanisms in the quantum spontaneous origin of the dissipative structures observed experimentally in water (xerosydryle). The same dynamic principle, termed “Like Likes Like” by Nobel laureate physicist Richard Feynman could explain the formation and remarkable stability of water’s supramolecular structures. Given the theoretically predicted and experimentally observed abundance of electrons, a correspondingly large number of water molecules must be ionized, becoming positively charged. These ionized molecules can attract each other and stabilize through spontaneous supramolecular organization, forming the observed dissipative structures. Moreover, these structures are so stable that, upon water drying or lyophilization, they condense into substantial quantities with each nucleus being hundreds of nanometers in size.

The fractal behavior of these dissipative structures was experimentally demonstrated in [1], which further confirms their coherent quantum nature. An isomorphism exists between the observed scale-free, self-similar properties of the modified liquid water and the deformed coherent state formalism. The fractal dimension measures a dynamic “deformation” indicating that the observed scale-free law relating pH and electric conductivity is a macroscopic manifestation of dissipative local deformations at a microscopic level. This intriguing isomorphism was theoretically identified in its entirety in a seminal work by G. Vitiello [97], which demonstrated that fractal self-similarity properties can be described using coherent states. Consequently, quantum dissipation, through quantum deformation or squeezing of coherent states, appears to underlie the self-similarity properties observed at a macroscopic level.

### Appendix A.4. Is There a Possibility of Contamination? How Many Impurities (That May Result from the Processing of the Liquid Phase) Can Be Ruled Out?

It is helpful to begin by defining the context of our experimental findings from a broad perspective.

In this paper, we reported, for the first time, evidence of micron-sized chiral supramolecular H_2_O aggregates in water under ambient conditions: [60]. These aggregates were generated by physically perturbing pure water through a process involving the iterative immersion of a hydrophilic membrane (Nafion^®^) in Milli-Q^®^ water, followed by stirring, membrane removal, and drying. The circular dichroism spectra of such perturbed water exhibit similarities to β-sheet ordered biomolecules. Additionally, lyophilization of the perturbed water resulted in the formation of a solid residue. To rule out the possibility that impurities (released by the membrane), organic or bio-contaminants were causing these phenomena, we employed advanced analytical techniques, such as Matrix-Assisted Laser Desorption/Ionization Time of Flight (MALDI-TOF) spectroscopy, Gas Chromatography coupled with Mass Spectroscopy (GC-MS), and Ion Chromatography. The “iteratively nafionated water” was found to contain only 10^−6^ M fluorine and sulfate ions released by the membrane, as documented on page 29 in Table S1 of [60]. It also showed a negligible amount of contaminants. Furthermore, we demonstrated that the UV absorbance and fluorescence spectra of the “iteratively nafionated water” could not be attributed to contaminants or molecules released by the membranes.

Our findings illustrated that aggregates forming in water adjacent to a hydrophilic material can assume and retain a stable chiral configuration. Several months following the publication of our study, this discovery was substantiated by the identification of DNA’s chiral spine of hydration [98], marking a notable progression in comprehending water properties and their significant implications for biological science. The findings detailed in the following paper demonstrate how water near DNA, mirroring our observations with other hydrophilic substances, exhibits flexibility and the capacity to conform to a resilient supramolecular structure aligned with DNA’s chirality.

There is a fundamental consideration that we believe to be essential: in some cases, when certain types of hydrophilic materials come into contact with pure water, we observe the formation of up to 6 g/L of xerosydryle. These water supramolecular aggregates represent a solid residue resulting from the interaction between the material and water. To put this into perspective, consider a 1-m-side cubic aquarium filled with double-distilled water. The intriguing question arises: How could one obtain 6 kg of bacteria or other contaminants within just a few hours? The magnitude of this phenomenon is truly remarkable.

Finally, there is a crucial epistemological consideration that holds significant importance from our perspective. Throughout the same type of experimentation, namely ‘iterative perturbation of pure water’, we observe:

- consistently reproducible results across various chemical and physical characteristics of water;
- uniform behavior across different insoluble materials used to perturb water (e.g., an increase in electric conductivity with the number of procedure iterations).

However, we also report specific outcomes depending on the type of perturbing material used (e.g., a significant alkaline shift in pH in some cases, a strong acidic shift in another, while remaining neutral in others).

These observations suggest the insignificance of any potential interfering factors on the overarching phenomenon identified.

Typically, an increase in χ necessitates the addition of an electrolyte to water, and an increase in pH requires the addition of a basic substance. However, in our case, this new phenomenology reaches a plateau after a certain time interval, indicating that, akin to low-solubility salts, a maximum concentration of water structures is achieved at the plateau value. The phenomenon remains reproducible even when the same container is reused in subsequent experiments, discrediting the hypothesis of impurity release from the container. The synchronous behavior of the two parameters is fundamental.

### Appendix A.5. Is There a Possibility That Xerosydryle and Biological Objects May Interact in One Way or Another?

In our papers, we briefly discussed the biological implications of the existence and discovery of xerosydryle, suggesting that it can contribute to our understanding of the ‘matrix of life’ itself. When xerosydryle is dissolved in water, it particularly exhibits circular dichroism. Future research, which will not be easy to perform, will focus on determining whether right- or left-handed chirality is preferred under certain conditions. This may provide insights into why almost all biologically produced chiral amino acids are left-handed, or into the dominance of the right-handed B-form of DNA.

Interestingly, our measurements show that, when dissolved in water, the thermal properties of xerosydryle resemble those of biological macromolecules (commonly referred to as ‘denaturation’). However, xerosydryle is more thermally resistant than biomolecules and, in this study, its chirality was observed to be unaffected by the addition of sodium hydroxide (NaOH) or hydrogen chloride (HCl) in sufficient amounts to raise the pH to 13 or lower it to 3, respectively. Such robustness, along with the change in the hydration state, can lead to dramatic changes in the DNA structure and may be related to one of the DNA repair mechanisms. Generally, the origin of biohomochirality remains inexplicable. Our data suggest that the mirror-symmetry breaking effect in the water may significantly contribute to the origin and prevalence of biohomochirality. For future research, it will be very important to investigate the impact of chiral supramolecular H_2_O aggregates on prebiotic chemical reactions and abiogenesis processes. The dynamic processes by which these dissipative structures are stabilized, and the ability of these structures to recreate their previous physicochemical properties when placed back into pure water, is reminiscent of the ability of some simple living systems, such as bacteria or protists, to remain in a quiescent state when environmental conditions are unfavorable to life. The process of encystment helps the microbe survive until conditions become more favorable. When the encysted microbe finds an environment conducive to its growth and survival, the cyst wall breaks down (excystation). Specifically, when sufficient water is present, the microbe returns to its characteristic far-from-equilibrium state, that of a dissipative, living structure.
