# Supplementary material for: Hints of Biological Activity of Xerosydryle: Preliminary Evidence on the Early Stages of Seedling Development
Source: Int J Mol Sci. 2024 Aug 9;25(16):8717. doi: 10.3390/ijms25168717 (PMC11354744; doi:10.3390/ijms25168717)
Supplement: Supplementary file 1 [file ijms-25-08717-s001.zip › Supplementary Figure S1.pdf]

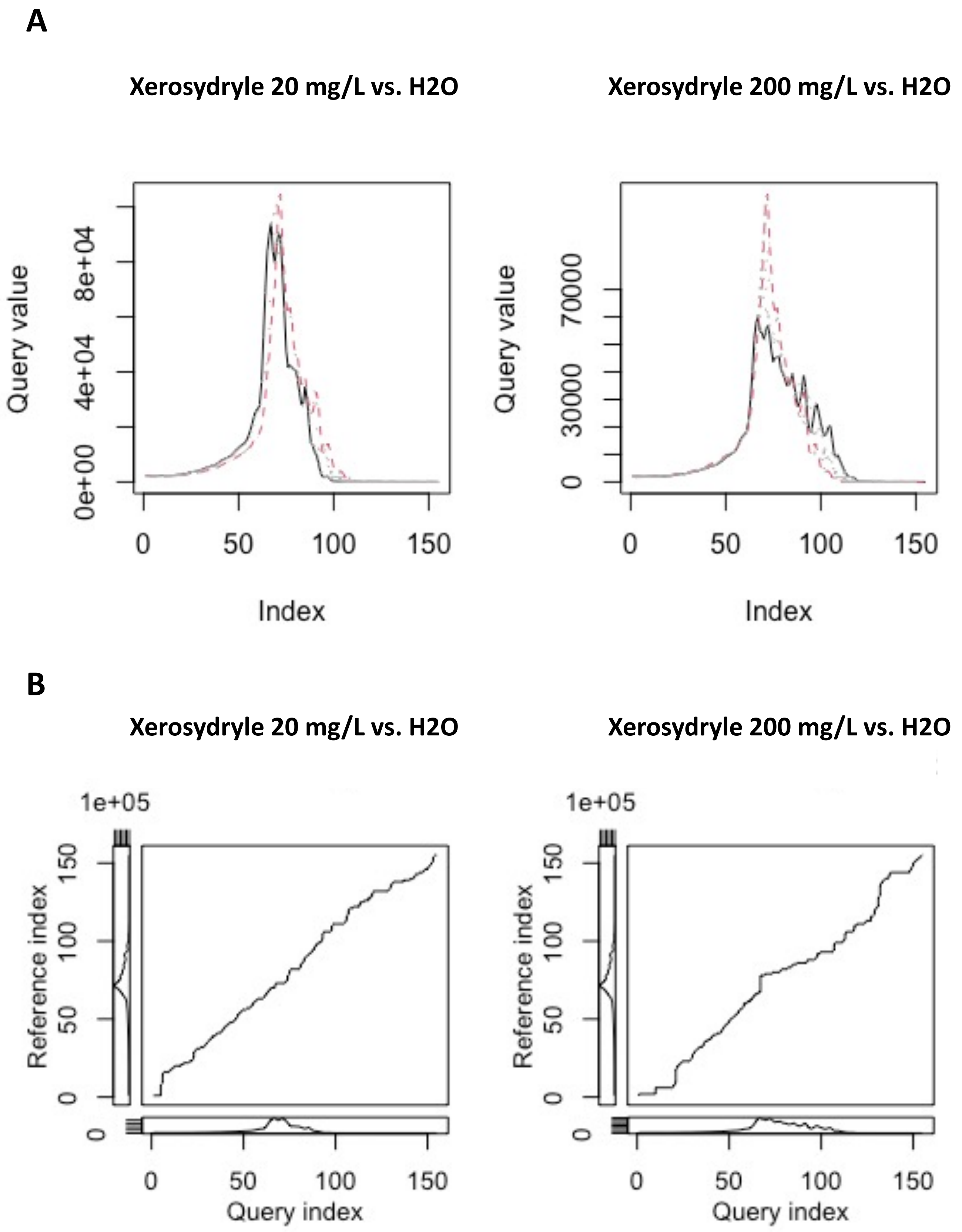

Supplementary Figure S1. Dynamic Time Warping (DTW) analysis of chlorophyll spectra. (**A**) plots of Xerosydryle 20 and 200 mg/L vs. H2O; (B) Time series alignment of Xerosydryle 20 and 200 mg/L vs. H2O
