# Supplementary material for: Hints of Biological Activity of Xerosydryle: Preliminary Evidence on the Early Stages of Seedling Development
Source: Int J Mol Sci. 2024 Aug 9;25(16):8717. doi: 10.3390/ijms25168717 (PMC11354744; doi:10.3390/ijms25168717)
Supplement: Supplementary file 1 [file ijms-25-08717-s001.zip › Supplementary Figure S2.pdf]

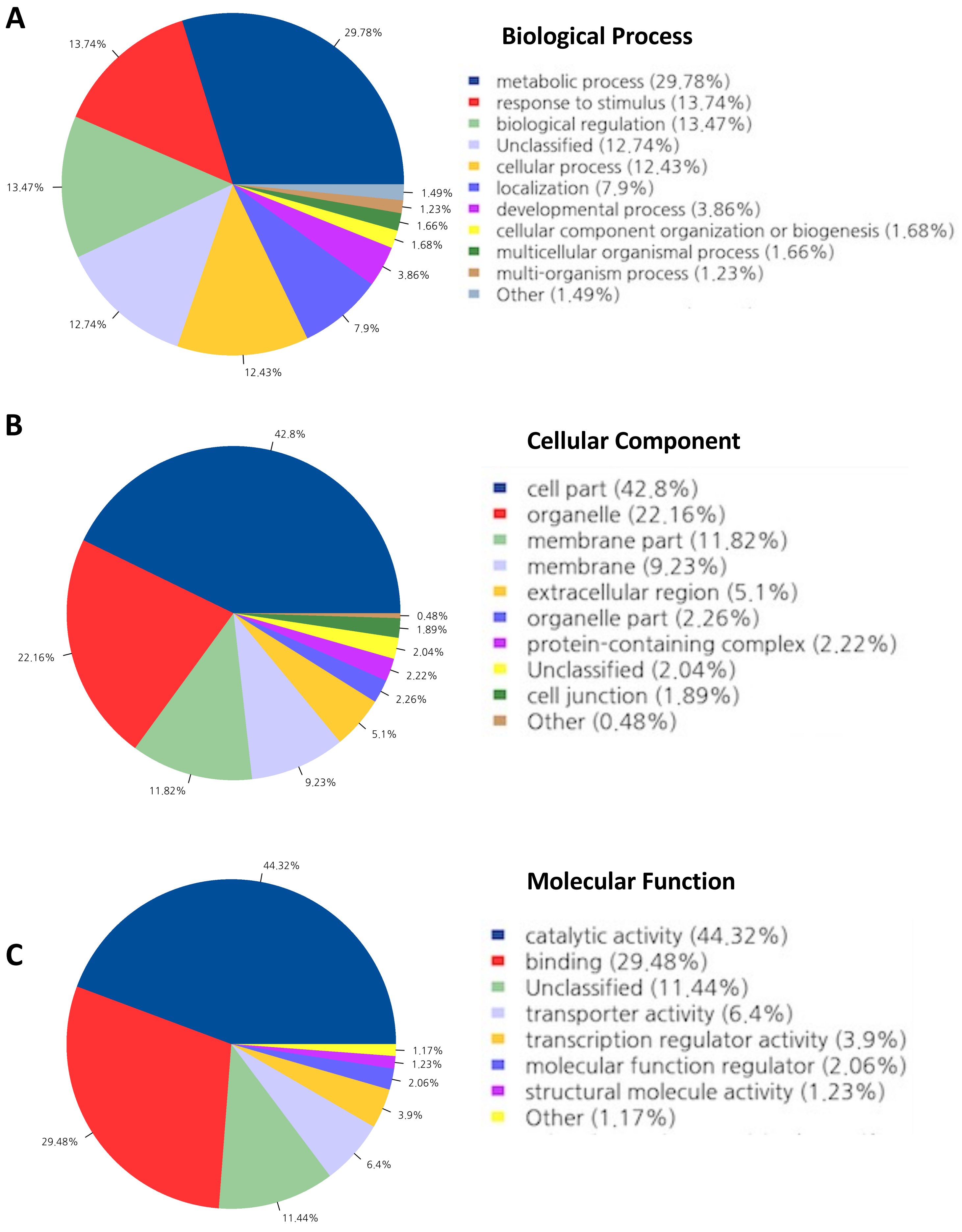

Supplementary Figure S2. Gene Ontology (GO) descriptors. Differentially expressed transcripts are classified according to (**A**) 'Biological Process'; (**B**) 'Cellular Components'; (**C**) 'Molecular Function'.
